# Assessing the Impact of Video Games on Social Interactions and Isolation Among Male Students in Various Universities in Riyadh, Saudi Arabia

**DOI:** 10.7759/cureus.96531

**Published:** 2025-11-10

**Authors:** Layan D Alshehri, Nawaf Alnuwaysir, Kady Alsarhan, Amaal M AlQahtani, Aryam F Almutairi, Nouf A Zurayyir, Maha A Alanazi, Rola Alasmari, Leen D Alajmi, Jana A Alhulayyil, Shahad Z Alsharif, Dalia W Alqahtani, Lujain M Alwadani

**Affiliations:** 1 College of Health and Rehabilitation Sciences/Epidemiology, Princess Nourah Bint Abdulrahman University, Riyadh, SAU; 2 Community Health Sciences Department, King Saud University, Riyadh, SAU

**Keywords:** internet gaming disorder, loneliness, male students, saudi arabia, social isolation, video gaming

## Abstract

Background and objective

Video gaming (VG) has become a global phenomenon. Prolonged gaming can lead to a loss of control and prioritization of gaming over daily activities, a condition known as gaming disorder, which may reduce social interactions and influence how individuals relate to others. This study aimed to evaluate awareness and perceptions of the effects of video gaming on mental well-being. Additionally, it sought to examine the duration and frequency of gaming and their association with the severity of social isolation and loneliness.

Methods

We conducted a cross-sectional, questionnaire-based study involving 1,279 male students across different universities in Riyadh. The questionnaire was organized into five sections, covering areas such as sociodemographic characteristics and knowledge regarding the effects of gaming on social isolation. Data were collected for the period from October 2024 until April 2025.

Results

A total of 1,071 students reported playing video games, with 52.85% acknowledging that video game players tend to be socially isolated. The majority of participants were from Science colleges (23.25%), and approximately 42.86% reported spending two to four hours per day gaming. Computers and tablets emerged as the most commonly used devices (41.13%). Notably, 69% of students were aware of the negative impact of video gaming on social interactions. A significant association was observed between the number of daily gaming hours and the level of social isolation (p<0.0001).

Conclusions

The study emphasizes the need for public health intervention that promotes a more balanced relationship between video gaming and real-life engagement. It recommends collaboration between universities and policymakers to raise awareness of the potential risks associated with video gaming and to promote a healthier balance between digital activities and in-person social interaction.

## Introduction

The rising popularity of video gaming (VG) may contribute to reduced social interactions and decreased participation in family or peer activities. As a global trend, video gaming has transformed how individuals of various ages engage in leisure and social connection. The World Health Organization (WHO) defines gaming disorder as a pattern of persistent or recurrent gaming behavior characterized by impaired control, prioritization of gaming over other daily activities, and continuation of play despite negative consequences [1]. A Saudi national study showed that 82.2% (n = 4385) of students play video games, with 20.45% meeting criteria for gaming disorder, ranging from 15.7% in Riyadh to 33.3% in Makkah [2]. Video gaming behavior is also shaped by younger age groups; for instance, one study found that among 393 Saudi youth aged 12-16, 63.1% played video games daily, and 25.2% spent more than five hours per day gaming. Additionally, a study at King Saud University in Riyadh revealed that 4.6% of 210 students were addicted to video gaming [4]. It is important to note that loneliness represents a significant public health concern, closely associated with depression and other psychological disorders [5].

Excessive engagement in video gaming may lead to diminished social interactions, including reduced participation in family gatherings, friendships, and community activities. Although several studies have examined the impact of video gaming in Saudi Arabia, a notable knowledge gap remains concerning the extent of social isolation experienced by male students across different universities in the country. Therefore, this study aims to (i) evaluate students’ awareness and perceptions of the impact of video gaming on social interactions, (ii) examine its influence on mental well-being, and (iii) analyze the relationship between the duration and frequency of gaming and the severity of social isolation and loneliness. By exploring these interconnected dimensions, the study seeks to generate evidence-based insights that can inform strategies to mitigate the adverse mental health effects of excessive gaming and promote healthier social engagement.

## Materials and methods

Study design, population, and sampling**

A cross-sectional study design was employed for this research. Convenience sampling was adopted to facilitate timely data collection and ensure accessibility to participants from multiple universities. This approach enabled the researchers to efficiently reach a diverse sample of male students representing various academic disciplines within Riyadh. The study population included male university students aged 18 years and older, with a total sample of 1,279 participants drawn from different universities across Riyadh.

Instrument

The questionnaire was developed by the authors based on an extensive review of the existing literature on gaming behavior, social interaction, and mental health, and was subsequently formatted and administered using Google Forms. The design of the tool was guided by the Knowledge, Attitudes, and Behavior (KAB) framework, which helped organize the questions into clear and meaningful categories. The questionnaire was designed to be completed in three to five minutes and comprised five sections. Section 1 collected sociodemographic information through four items, three of which were single-response and one allowing multiple responses (select-all-that-apply). Section 2 evaluated knowledge regarding the impact of gaming on social isolation using six items with three response options: “Yes,” “No,” or “I don’t know.” Section 3 assessed participants’ attitudinal and behavioral perceptions of gamer friends with a single item using the same response options. Section 4 included 10 items measuring agreement with statements about gaming and social isolation on a 5-point Likert scale ranging from “Strongly Agree” to “Strongly Disagree.” Section 5 examined gaming- and isolation-related behaviors through 13 items on a 4-point Likert scale ranging from “Always” to “Never.” Overall, the questionnaire contained 34 items, and all responses were securely stored in a digital database, facilitating efficient access for analysis.

Reliability and validity**

To assess the validity and reliability of the questionnaire, a pilot study involving 133 students was conducted before the main data collection to examine the clarity, comprehension, and usability of the items. To evaluate the reliability, Cronbach’s Alpha was calculated using SPSS Statistics, yielding a score of 0.70, which indicates an acceptable level of internal consistency. The feedback from the pilot study was then used to refine the questionnaire before full data collection.

Data collection

A self-administered, researcher-developed questionnaire was used to collect data for this study from October 2024 to April 2025. Students were invited to participate in the survey, which was distributed via social media platforms, including X, WhatsApp, and Telegram.

Inclusion and exclusion criteria**

Participants were deemed eligible if they were 18 years or older, engaged in regular video gaming (at least once per week), and provided voluntary consent to participate. A total of 1,279 responses were initially collected. Of these, 208 were excluded from the analysis: 133 responses were from participants in the pilot study, three lacked informed consent, 61 respondents reported not playing video games, and 11 were under 18 years of age. Consequently, a total of 1,071 responses met the inclusion criteria and were retained for the final analysis.

Statistical analysis

The dataset was translated from Arabic to English by a bilingual expert and subsequently cleaned to remove excluded responses. Descriptive statistics (frequencies, percentages, means, and standard deviations (SD) were used to summarize participant characteristics. Chi-square tests were performed to examine associations between categorical variables, with Cramer’s V and odds ratios (ORs) reported for effect size. Multiple-response questions were analyzed using frequency and percentage distributions. Attitudinal items were assessed using mean scores on a 5-point Likert scale, and composite indices were created for key constructs such as isolation level. Group differences in awareness of isolation and isolation score were assessed using one-way ANOVA. All analyses were conducted using JMP Pro 18 and Microsoft Excel.

Ethical considerations

The study received ethical approval from Princess Nourah Bint Abdulrahman University Institutional Review Board (IRB) under log number (24-0854). The initial IRB approval to conduct data collection was obtained in October 2024, while the final IRB approval for publication was granted after the completion of the study in October 2025. The study adhered to the guidelines of the Declaration of Helsinki. All participants provided informed consent before participation and were informed about the research purpose, participation requirements, and their right to withdraw at any time without repercussions. All collected data were used solely for research purposes, with participant confidentiality strictly maintained.

## Results

Table 1 summarizes the sociodemographic characteristics of the 1,071 male university student gamers surveyed. The majority of participants were aged 25-29 years (43.88%), followed by those aged 18-24 years (37.82%). A smaller proportion were aged 30 years and above (18.30%). In terms of college type, most participants attended science colleges (23.25%) and humanities colleges (19.61%), while health colleges had the lowest representation (8.12%). Regarding gaming duration, most respondents played for two to four hours per day (42.86%), followed by those who played for five to seven hours (32.40%). A smaller proportion reported playing for an hour or less (14.66%) or more than eight hours daily (10.08%).

**Table 1:** Sociodemographic characteristics of male university student gamers (N = 1,071)

Characteristic	N	%
Age group, years		
18-24	405	37.82%
25-29	470	43.88%
30 and above	196	18.30%
College currently attending		
Science college	249	23.25%
Humanities college	210	19.61%
Administrative college	207	19.33%
Engineering college	170	15.87%
Technical college	148	13.82%
Health college	87	8.12%
Number of hours playing video games per day		
1 or less	157	14.66%
2-4	459	42.86%
5-7	347	32.40%
≥8	108	10.08%

Table 2 shows the distribution of devices used for gaming. Computers or tablets were the most commonly used (41.13%), followed by gaming consoles such as PlayStation or Xbox (34.71%) and mobile phones (23.01%). Other devices accounted for only 1.15% of responses.

**Table 2:** Distribution of devices used for gaming (multiple-response question)

Variable	N	%
Mobile phone	301	23.01%
Computer or tablet devices	538	41.13%
Gaming consoles (PlayStation, Xbox, etc.)	454	34.71%
Other	15	1.15%
total	1308	100%

Table 3 summarizes participants’ knowledge about the effects of gaming on social isolation. The highest level of awareness was observed for the statement that video game addiction can negatively affect mental health due to reduced social interaction (69.28% responded “Yes”). In contrast, only 31.56% agreed that gaming cannot fully replace face-to-face interaction. Awareness that excessive gaming may harm family and social relationships was moderate (53.97%), and 37.82% recognized loneliness as a potential consequence of prolonged gaming. Approximately half (49.39%) believed that not participating in social activities due to gaming could lead to isolation. 

**Table 3:** Knowledge regarding the effects of gaming on social isolation

Question	Yes		No		I don’t know	
	N	%	N	%	N	%
Did you know that video game addiction can negatively impact mental health due to a lack of social interaction?	742	69.28%	108	10.08%	221	20.63%
Did you know that gaming is not a complete substitute for face-to-face social interaction?	338	31.56%	434	40.52%	299	27.92%
Did you know that excessive gaming involvement can lead to the deterioration of family and social relationships?	578	53.97%	213	19.89%	280	26.14%
Did you know that one of the psychological consequences of spending too much time playing video games is loneliness?	405	37.82%	335	31.28%	331	30.91%
Do you think not participating in social activities because of video games can lead to isolation?	529	49.39%	265	24.74%	277	25.86%
Did you know that video games can lead to isolation due to a lack of social interaction?	460	42.95%	312	28.13%	299	27.92%

**Figure 1:**
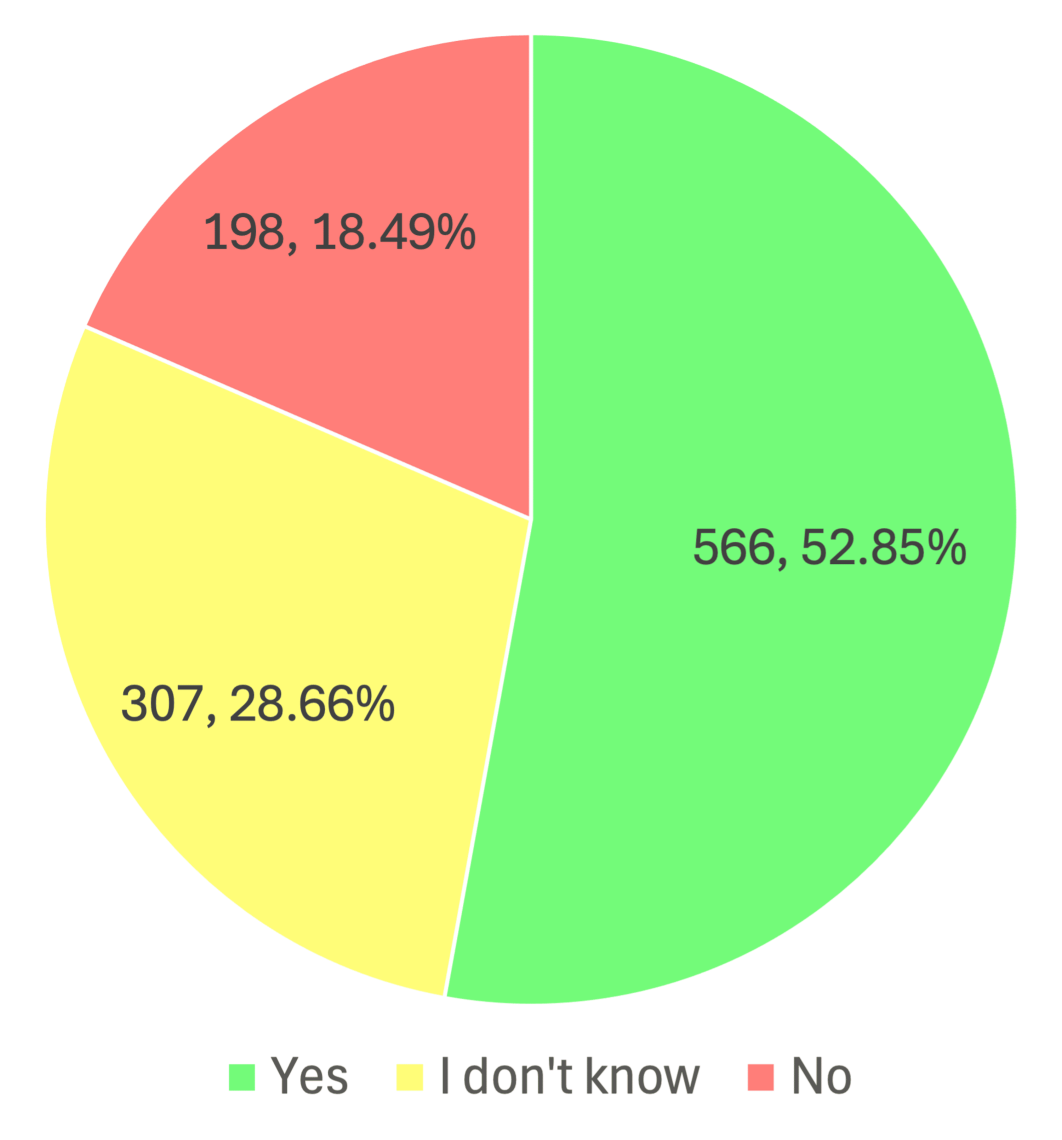
Attitudinal and behavioral perceptions of gamer friends

Figure 1 illustrates the distribution of responses to the question, “Have you noticed your gamer friends becoming isolated or preferring gaming over face-to-face conversations?” (N = 1,071). Over half of respondents (n = 566, 52.85%) reported noticing such behaviors among their gamer friends. In contrast, 18.49% (n = 198) indicated that they had not observed this pattern, while 28.66% (n = 307) stated that they were unsure. This distribution suggests that a majority of participants perceive a link between gaming and increased social isolation or reduced face-to-face interaction among their peers (Figure 1).

Table 4 outlines the levels of agreement with statements on gaming and social isolation. The highest agreement score (mean = 3.65, SD = 1.17) was for “Video games can lead to social isolation.” Agreement was also noted for “Video games are a way to avoid social interaction” (mean = 3.41, SD = 1.33). Other statements were rated as “Neutral,” including the relationship between time spent gaming and poor social interaction (mean = 3.13, SD = 1.32) and the effect of excessive gaming on social relationships (mean = 3.17, SD = 1.36).

**Table 4:** Level of agreement with statements on gaming and social isolation SD: standard deviation

Statement	Mean	SD	Interpretation
How much do you agree with the statement: “Video games can lead to social isolation”?	3.65	1.17	Agreement
How much do you agree with the statement: "Video games are a way to avoid social interaction"?	3.41	1.33	Agreement
How much do you agree with the statement: "It's good to spend a lot of time playing video games"?	3.22	1.29	Neutral
How much do you agree with the statement: "There is a relationship between time spent playing video games and poor social interaction"?	3.13	1.32	Neutral
How much do you agree with the statement: “Excessive time spent playing video games reduces the opportunity to interact with others face-to-face”?	3.36	1.33	Neutral
How much do you agree with the statement: “Excessive video game play negatively affects social relationships”?	3.17	1.36	Neutral
How much do you agree with the statement: “There should be a balance between social life and playing video games”?	3.33	1.36	Neutral
How much do you agree with the statement: “Taking long breaks from gaming improves social relationships”?	3.25	1.29	Neutral
How much do you agree with the statement: “Social isolation is a natural consequence of video game addiction"؟	3.13	1.32	Neutral
How much do you agree with the statement: “Video games can cause a feeling of isolation even when playing with others online"?	3.34	1.26	Neutral

Table 5 presents behaviors related to gaming and social isolation. Nearly one-third (29.60%) reported always choosing gaming over time with friends or family, while 23.06% always played more when feeling lonely. Social interactions within games were always considered a substitute for real-life interactions by 23.72% of respondents. Only 20.45% reported always attempting to reduce gaming time to improve social life, and 21.85% indicated such attempts were always successful. Additionally, 16.43% of participants reported "always" avoiding in-person gatherings in favor of gaming.

**Table 5:** Frequency of gaming and social isolation-related behaviors

Question	Always		Sometimes		Rarely		Never	
	N	%	N	%	N	%	N	%
Do you find yourself choosing to play video games instead of spending time with friends or family?	317	29.60%	447	41.74%	195	18.21%	112	10.46%
When you feel lonely, do you tend to play more video games to cope with those feelings?	247	23.06%	288	26.89%	274	25.58%	262	24.46%
Do you think social interactions within games can be a substitute for real-world social interactions?	254	23.72%	360	33.61%	216	20.17%	241	22.50%
Are you concerned about the time you spend gaming and how it affects your social relationships?	177	16.53%	317	29.60%	273	25.49%	304	28.38%
Have you ever tried cutting down on gaming time to improve your social life?	219	20.45%	379	35.39%	256	23.90%	217	20.26%
If so, did it work? (See previous question)	234	21.85%	325	30.35%	262	24.46%	250	23.34%
How likely are you to choose gaming over face-to-face conversations when you have free time?	212	19.79%	360	33.61%	270	25.21%	229	21.38%
Does the amount of time you play daily affect your desire to interact with others?	196	18.30%	373	34.83%	270	25.21%	229	21.66%
Do you find it difficult to participate in university social activities due to playing too many video games?	189	17.65%	353	32.96%	275	25.68%	254	23.72%
Do you tend to avoid in-person social gatherings in favor of staying home to play video games?	176	16.43%	371	34.64%	280	26.14%	244	22.78%
Have you noticed a decrease in your real-life social circle as a result of spending more time gaming?	204	19.05%	341	31.84%	288	26.89%	238	22.22%
Do you think video games are your only way to communicate with others?	180	16.81%	339	31.65%	256	23.90%	296	27.64%
Have you lost some of your social connections due to your immersion/interest in games?	129	12.04%	418	39.03%	326	30.44%	198	18.49%

Table 6 shows the association between daily gaming hours and attempts to reduce gaming to improve social life. Participants who played for two to four hours per day constituted the largest group consistently attempting to cut back (39.73%), compared with only 10.96% among those who played for one hour or less. This association was statistically significant (χ²(9, N = 1,071) = 20.987, p = 0.0127 Cramer’s V = 0.081).

**Table 6:** Association between daily gaming hours and attempts to reduce gaming time to improve social life Percentages are calculated within each column and may not sum to exactly 100% due to rounding

Hours/day of gaming	Always, n (%)	Sometimes, n (%)	Rarely, n (%)	Never, n (%)	Total, n (%)
≤1	24 (10.96%)	72 (19.00%)	38 (14.84%)	23 (10.60%)	157 (14.66%)
2–4	87 (39.73%)	164 (43.27%)	116 (45.31%)	92 (42.92%)	459 (42.86%)
5–7	82 (37.44%)	105 (27.70%)	86 (33.59%)	74 (34.10%)	347 (32.40%)
≥8	26 (11.87%)	38 (10.03%)	16 (6.25%)	28 (12.90%)	108 (10.08%)
Total	219 (20.45%)	379 (35.39%)	256 (23.90%)	217 (20.26%)	1,071

Table 7 displays the association between daily gaming hours and the level of social isolation. High levels of isolation were most common among participants who played for five to seven hours per day (54.02%), followed by those gaming for eight hours or more (18.39%). Conversely, low isolation levels were more frequent among individuals who played for one hour or less (32.89%) or for two to four hours daily (50.00%). The association was statistically significant (χ²(6, N = 1,071) = 62.694, p<0.0001, Cramer’s V = 0.171). 

**Table 7:** Association between daily gaming hours and level of social isolation Percentages are calculated within each column and may not sum to exactly 100% due to rounding

Hours/day of gaming	High, n (%)	Moderate, n (%)	Low, n (%)	Total, n (%)
≤1	5 (5.75%)	127 (13.99%)	25 (32.89%)	157 (14.66%)
2–4	19 (21.84%)	402 (44.27%)	41 (50.00%)	459 (42.86%)
5–7	47 (54.02%)	290 (31.94%)	10 (13.16%)	347 (32.40%)
≥8	16 (18.39%)	89 (9.80%)	3 (3.95%)	108 (10.08%)
Total	87 (8.12%)	908 (84.78%)	76 (7.10%)	1,071

Table 8 provides odds ratios for selected social isolation-related outcomes. Awareness of isolation (OR = 0.44, 95% CI = 0.35-0.57, p<0.0001) and belief in balance between gaming and real life (OR = 0.51, 95% CI = 0.40-0.65, p<0.0001) were significantly associated with reduced odds of high isolation. No significant associations were found regarding friends’ preference (p = 0.2303) or social avoidance (p = 0.1188).

**Table 8:** Odds ratios for selected social isolation-related outcomes ^*^Statistically significant OR: odds ratio; CI: confidence interval

Outcome variable	OR	95% CI	P-value
Friends’ preference	0.82	0.59–1.14	0.2303
Awareness of isolation	0.44	0.35–0.57	<0.0001^*^
Belief in balance	0.51	0.40–0.65	<0.0001^*^
Social avoidance	1.21	0.95–1.55	0.1188

**Figure 2:**
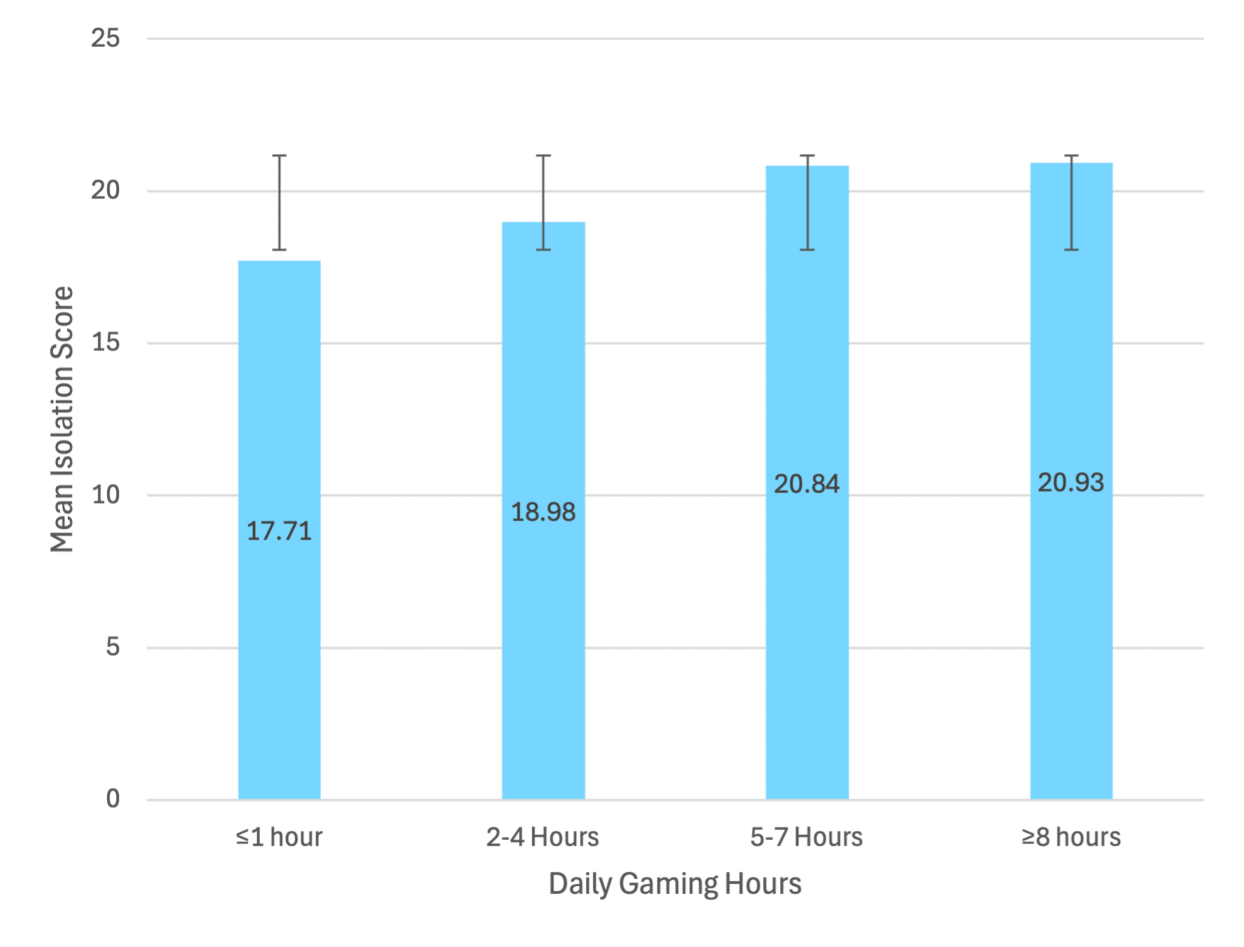
Mean isolation scores by daily gaming hours (N = 1,071) One-way ANOVA indicated a significant difference between groups, F(3, 1067) = 22.18, p<0.0001

Figure 2 illustrates the relationship between daily gaming hours and mean isolation scores among participants. A clear upward trend was observed, indicating that students who spent more time gaming reported higher levels of social isolation (p<0.0001).

**Figure 3:**
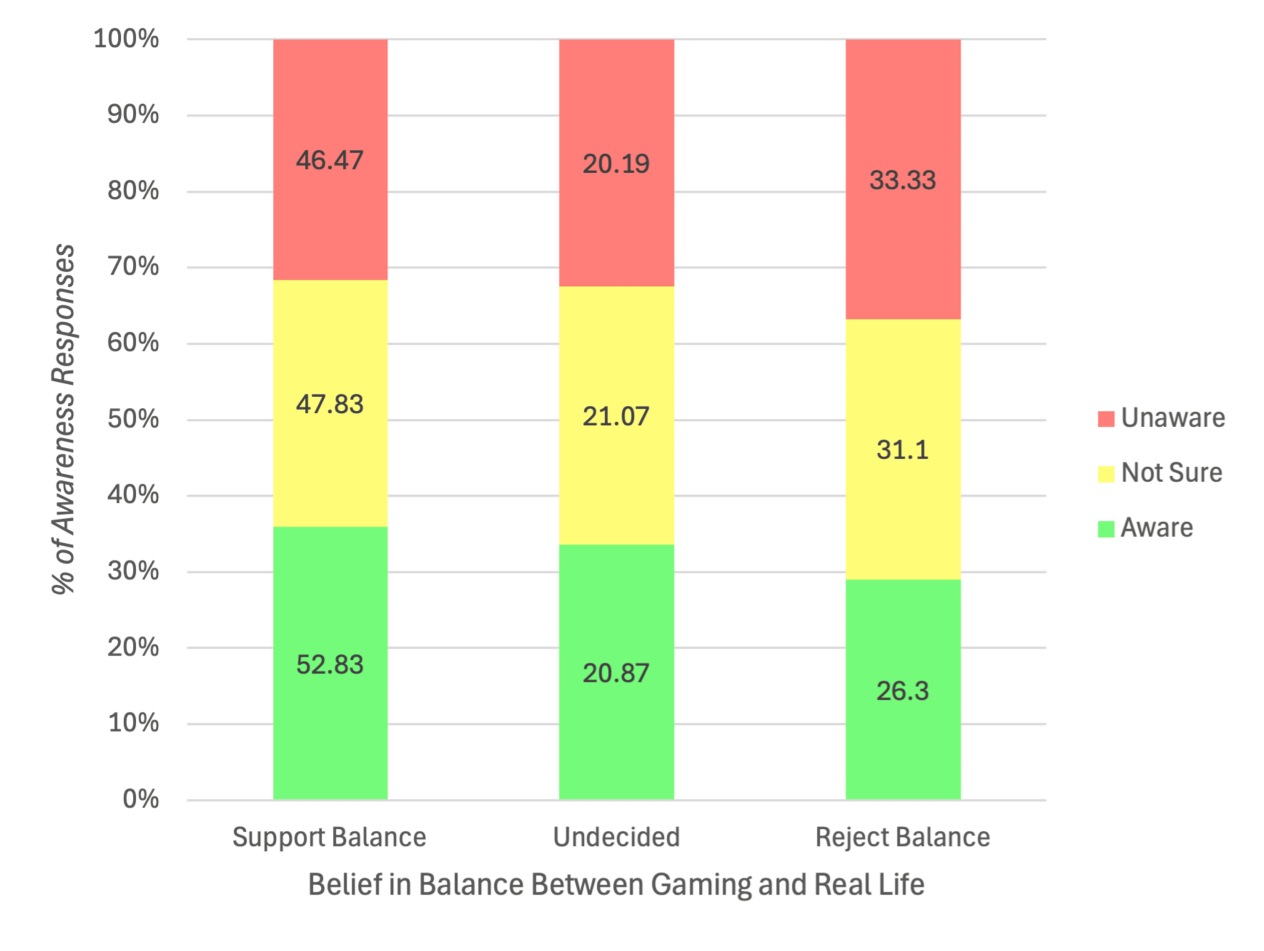
Relationship between awareness of isolation and belief in balance between gaming and real life (N = 1,071) Note: χ²(4, N = 1,071) = 5.21, p = 0.27

Figure 3 illustrates the relationship between participants’ awareness of social isolation and their belief in maintaining a balance between gaming and real life. The results show that those who were aware of the risks of isolation and supported balanced gaming exhibited lower isolation levels, suggesting that awareness and balanced attitudes may serve as protective factors.

Awareness of isolation and isolation scores represented in Table 9 did not differ significantly by college type (F(5,1065) = 1.94, p = 0.084; F(5,1065) = 1.81, p = 0.107) or age group (F(2,1068) = 2.71, p = 0.067; F(2,1068) = 1.71, p = 0.181). In contrast, daily gaming duration was significantly associated with both awareness and isolation levels, with higher gaming hours linked to lower awareness and higher isolation scores (F(3,1067) = 94.04, p<0.0001; F(3,1067) = 22.18, p<0.0001).

**Table 9:** Association of sociodemographic characteristics with awareness of isolation and isolation score ^*^Statistically significant Awareness of isolation reflects the total knowledge score regarding the risks and consequences of gaming-related isolation. The isolation score represents behavioral engagement and the perceived isolation level SD: standard deviation

Sociodemographic variable	Groups	Awareness, mean ± SD	Test (t/F)	P-value	Isolation score, mean ± SD	Test (t/F)	P-value
College	Science colleges	33.30 ± 5.50	F(5,1065)=1.94	0.084	19.22 ± 4.11	F(5, 1065) = 1.81	0.1072
	Humanities colleges	33.41 ± 6.53			19.43 ± 5.28		
	Administrative colleges	32.46 ± 6.25			20.4 ± 4.97		
	Engineering colleges	33.30 ± 5.73			19.17 ± 4.27		
	Technical colleges	31.89 ± 4.92			19.7 ± 4.71		
	Health colleges	33.46 ± 5.82			19.78 ± 6.05		
Age group, years	18-24	33.35 ± 6.34	F(2, 1068) = 2.71	0.067	19.27 ± 5.98	F(2, 1068) = 1.71	0.181
	25-29	32.51 ± 5.44			19.87 ± 3.8		
	30 and above	33.34 ± 5.79			19.61 ± 4.25		
Daily gaming hours	1 or less	36.72 ± 5.14	F(3, 1067) = 94.04	< .0001*	17.71 ± 5.09	F(3, 1067) = 22.18	<0.0001^*^
	2-4	34.69 ± 5.25			18.98 ± 4.63		
	5-7	29.95 ± 5.25			20.84 ± 4.59		
	More than 8	29.98 ± 5.17			20.93 ± 4.54		

## Discussion

Sociodemographic factors, gaming habits, and device preferences

The results indicated that the majority of students in the study were aged 25-29 years (n = 470, 43.88%), followed by those aged 18-24 years (n = 405, 37.82%). Students enrolled in science colleges represented the largest group (n = 249, 23.25%), followed by those from humanities and administrative colleges, while students from health colleges constituted the smallest group of gamers (n = 87, 8.12%). Similarly, A study from Thailand [6] found that 8.4% of medical students had internet gaming disorder (IGD), which was linked to depression. This may suggest that health students either have less free time to spend on gaming due to academic demands or are more conscious of the potential health risks linked to excessive gaming.

The findings show that most participants used computers or tablets (n = 538, 41.13%) and gaming consoles (n = 454, 34.71%), while fewer relied on mobile phones (n = 301, 23.01%). In contrast, a study involving 393 participants found that 74% of adolescents aged 12-16 years preferred mobile phones for gaming, compared to 21.6% who used tablets [3]. This perhaps shows a preference for computers and consoles in our participants that allows longer and more deeply involved play. Such choices matter because high-performance devices are often linked to extended sessions, which might increase gaming duration and therefore social isolation.

Regarding gaming duration, In terms of gaming duration, the majority of participants reported playing for two to four hours daily (n = 459, 42.86%), followed by those playing five to seven hours (n = 347, 32.40%), with the smallest group comprising students who played for more than eight hours per day. Similarly, adolescents aged 12-16 reported gaming for three to four hours daily (18.1%), with 25.2% playing more than five hours [3]. Surprisingly, one study [7] reported that among 726 students, approximately 74% played video games for <four hours on weekdays, while 71.5% played for <six hours on weekends. Moreover, as illustrated in Figure 1, more than half of the students (n = 566, 52.85%) reported noticing their gamer peers becoming more isolated or preferring gaming over direct conversations. A study from Abha [8] reported that many students preferred gaming over spending time with friends (n = 145, 53.5%) or family (n = 141, 52%), and experienced family troubles due to gaming (n = 96, 35.42%).

This finding indicates the importance of peers, friends, and families recognizing excessive gaming among others, as early awareness may allow them to suggest supportive interventions to help reduce gaming duration. Such recognition might also aid in identifying underlying mental health issues at an earlier stage. Overall, the high prevalence of video game use observed in our study underscores the importance of public health and community engagement to raise awareness, promote healthier gaming habits, and develop interventions that mitigate the risks of social isolation.

Awareness of social isolation risks

Our results show mixed levels of awareness about the social effects of gaming. Most students (n = 742, 69.28%) acknowledged the risks of gaming to mental health, while 31.56% (n = 338) recognized that gaming cannot substitute for face-to-face interaction. Slightly over half (n = 578, 53.97%) perceived potential negative effects on family and social relationships, whereas fewer students associated gaming with feelings of loneliness (n = 405, 37.82%) or social isolation resulting from avoiding activities (n = 529, 49.39%). Interestingly, one study [9] reported that individuals with IGD had significantly higher levels of social phobia. Moreover, a study from Abha city [8] found that key motivators for gaming were stress and anxiety relief (n = 161, 59.4%) and increased self-esteem (n = 116, 42.8%). Playing video games may serve as a way to escape from reality and stress, which could explain the link between gaming duration and loneliness.

A cross-sectional study [10] of 276 students in international schools in Buraydah found that 16% were addicted to video games, with a strong association between addiction and psychological distress. Another study [11] found that 11.4% (n = 638) of Chinese adolescents and young adults reported escapism as a primary gaming motivation across both genders. One study [12] examined 548 Indian participants aged 15-24 years and found that 18.8% met criteria for IGD, with prevalence higher among males. These studies indicate that a significant portion of young people are at risk of gaming-related problems worldwide, emphasizing the importance of addressing this issue.

Attitudes toward gaming and social interaction, and predictors of social isolation outcomes

Our findings showed that students largely agreed that video games can lead to social isolation (mean = 3.65, SD = 1.17) and may serve as a way to avoid direct social interaction (mean = 3.41, SD = 1.33). Odds ratio analysis indicated that students who were aware of the risks of isolation (OR = 0.44, 95% CI = 0.35, 0.57, p<0.0001) and those who valued a balance between gaming and real life (OR = 0.51, 95% CI = 0.40, 0.65, p<0.0001) had significantly lower odds of reporting high levels of isolation. Such results underscore the importance of preventive interventions that not only raise awareness but also promote moderation in gaming behaviors.

Behavioral patterns related to gaming and isolation

According to the findings, 29.60% of students (n = 317) reported always choosing gaming over spending time with friends or family, while 23.06% (n = 247) indicated they turned to gaming when feeling lonely. Nearly 20% of respondents (n = 212) felt that gaming could replace face-to-face interactions, demonstrating how gaming has changed from being largely used for pleasure to being a coping strategy for social and emotional stress. These results align with a study [13] conducted during the COVID-19 pandemic, which found a positive correlation between social isolation and IGD.

In our study, 16.53% of participants (n = 177) reported “always” and 29.60% (n = 317) reported “sometimes” being concerned about the impact of gaming on their relationships, while only 21.85% (n = 234) consistently succeeded in reducing their gaming time. This reflects students' addiction to video games and their inability to control this behavior, which might lead to a weakening of real-life social bonds over time.

Relationship between gaming duration and social isolation

Our results showed a significant association between daily gaming duration and attempts to reduce gaming. The gamers who play two to four hours daily are more likely to always attempt a reduction (n = 87, 39.73%) compared to those playing ≥8 hours (n = 26, 11.87%). This finding suggests that some players are aware of the negative impact of excessive gaming on their social life and attempt to reduce their gaming. In addition, daily gaming hours were also associated with levels of social isolation. The highest level of isolation was observed among players gaming five to seven hours daily (n = 47, 54.02%). One study from Saudi Arabia [3] reported significant positive correlations between gaming addiction and attention deficit hyperactivity disorder (ADHD) (r = 0.527), anxiety (r = 0.366), and depression (r = 0.321).

Strengths and limitations

This study has several notable strengths. First, the relatively large sample of 1,071 male university students from universities across Riyadh ensured adequate representativeness. Second, data were gathered using a structured questionnaire based on the KAB framework, enabling a systematic assessment of gaming behaviors and social interactions. Moreover, the use of descriptive and inferential statistics offered a comprehensive understanding of a possible relationship between gaming behavior and social isolation. Finally, this study addressed a timely and underexplored issue in the Saudi context, filling a knowledge gap and providing evidence that can inform preventive strategies, interventions, and awareness campaigns for university students.

On the other hand, this paper has several limitations that should be considered when interpreting the results. First, the cross-sectional design provides only a snapshot in time, which limits the ability to establish causal relationships between gaming habits and social isolation. Second, the use of convenience sampling limits the generalizability of the findings, as the sample may not fully represent all university students in Riyadh or other regions of Saudi Arabia. Third, the study included only male participants, thereby excluding female perspectives and potentially limiting broader applicability. Moreover, the reliance on self-reported questionnaires introduces the possibility of reporting biases, such as recall bias, or under- and over-reporting of behaviors. In addition, since the sample was drawn exclusively from universities in Riyadh, the results may not fully reflect the experiences of students in other regions of Saudi Arabia with different cultural or social contexts. Finally, the study did not comprehensively control for other potential confounders, such as academic stress, socioeconomic status, or personality traits, which may also influence social isolation.

Implications for future research and recommendations

This study highlights the need for universities/policy makers to promote awareness about the risks of excessive gaming and encourage a healthier balance between digital engagement and face-to-face interaction. Counseling services should integrate gaming-related concerns into student support programs, while families and communities can play a role in fostering alternative social activities to reduce isolation. Future research should include female participants, employ longitudinal designs to clarify causal relationships, and examine different regions of Saudi Arabia to gain a deeper understanding of cultural influences on gaming and social isolation.

## Conclusions

This study identified a possible association between excessive video gaming and increased social isolation among male university students in Riyadh, Saudi Arabia. Although most participants were aware of the potential mental health risks linked to prolonged gaming, many underestimated its broader social implications. These findings emphasize the importance of preventive strategies and awareness initiatives aimed at promoting balanced digital engagement and reducing the risk of social isolation among university students. It highlights the need for coordinated efforts between universities, public health institutions, and policymakers to design and implement interventions, such as health education/promotion, that encourage moderation, strengthen real-world social connections, and mitigate gaming-related behavioral risks. Future research should include female participants, adopt other study designs to explore causal relationships, and examine additional psychosocial variables such as stress, academic performance, and family relationships to better understand the underlying mechanisms linking gaming behavior with social isolation and mental health outcomes.

## Appendices

**Table 10:** Questionnaire items in English

Sections	Questions with answers
Section 1: Sociodemographic Characteristics (4 questions)	College currently attending: 1 - health colleges, 2 – science colleges, 3 - administration colleges, 4 - humanities colleges, 5 - engineering colleges, 4 - technical Colleges
	Age: 1 - less than 18 years old, 2 - 18-24 years old, 3 - 25-29 years old, 4 - 30 years old and above
	Number of hours playing video games per day: 1 - an hour or less, 2 - 2-4 hours, 3 - 5-7 Hours, 4 - more than 8 hours
	Type of devices used to play video games: 1 - mobile phone, 2 - computer or tablet devices, 3 - gaming consoles (PlayStation, Xbox, etc.), 4 - other
Section 2: Knowledge Regarding the Effects of Gaming on Social Isolation (7 questions / yes, no, I don’t know)	Did you know that video game addiction can negatively impact mental health due to a lack of social interaction?
	Have you noticed that your peers who play video games tend to be isolated or prefer gaming over face-to-face conversations?
	Did you know that gaming is not a complete substitute for face-to-face social interaction?
	Did you know that excessive gaming involvement can lead to the deterioration of family and social relationships?
	Did you know that one of the psychological consequences of spending too much time playing video games is loneliness?
	Do you think not participating in social activities because of video games can lead to isolation?
	Did you know that video games can lead to isolation due to a lack of social interaction?
Section 3: Level of Agreement Toward Gaming and Social Isolation Statements (10 questions; 5-point Likert scale: Strongly Agree → Strongly Disagree)	How much do you agree with the statement: “Video games can lead to social isolation”?
	How much do you agree with the statement: "Video games are a way to avoid social interaction"?
	How much do you agree with the statement: "It's good to spend a lot of time playing video games"?
	How much do you agree with the statement: "There is a relationship between time spent playing video games and poor social interaction"?
	How much do you agree with the statement: “Excessive time spent playing video games reduces the opportunity to interact with others face-to-face”?
	How much do you agree with the statement: “Excessive video game play negatively affects social relationships"?
	How much do you agree with the statement: “There should be a balance between social life and playing video games"?
	How much do you agree with the statement: “Taking long breaks from gaming improves social relationships"?
	How much do you agree with the statement: “Social isolation is a natural consequence of video game addiction"?
	How much do you agree with the statement: “Video games can cause a feeling of isolation even when playing with others online"?
Section 4: Gaming and Social Isolation-Related Behaviors (13 questions; 4-point Likert scale: Always → Sometimes → Rarely → Never)	Do you find yourself choosing to play video games instead of spending time with friends or family?
	When you feel lonely, do you tend to play more video games to cope with those feelings?
	Do you think social interactions within games can be a substitute for real-world social interactions?
	Are you concerned about the time you spend gaming and how it affects your social relationships?
	Have you ever tried cutting down on gaming time to improve your social life?
	If so, did it work? (See previous question)
	How likely are you to choose gaming over face-to-face conversations when you have free time?
	Does the amount of time you play daily affect your desire to interact with others?
	Do you find it difficult to participate in university social activities due to playing too many video games?
	Do you tend to avoid in-person social gatherings in favor of staying home to play video games?
	Have you noticed a decrease in your real-life social circle as a result of spending more time gaming?
	Do you think video games are your only way to communicate with others?
	Have you lost some of your social connections due to your immersion/interest in games?

**Table 11:** Questionnaire items in Arabic

الأسئلة	القسم
الكلية التي تدرس فيها حاليًا : • الكليات الصحية • الكليات الهندسية • الكليات الإنسانية • الكليات الإدارية • الكليات العلمية • الكليات التقنية	القسم الأول: الخصائص الديموغرافية (4 أسئلة)
العمر: • أقل من 18 سنة • من 18 إلى 24 سنة • من 25 إلى 29 سنة • 30 سنة فأكثر	
عدد الساعات اليومية المخصصة لألعاب الفيديو: • ساعة أو أقل • من ساعتين إلى 4 ساعات • من 5 إلى 7 ساعات • أكثر من 8 ساعات	
الأجهزة المستخدمة في لعب ألعاب الفيديو (يمكن اختيار أكثر من خيار): • الهاتف المحمول • الكمبيوتر أو الأجهزة اللوحية • أجهزة الألعاب (مثل البلايستيشن، الإكس بوكس، وغيرها) • أخرى	
هل لديك معرفة بأن إدمان ألعاب الفيديو قد يؤثر سلبًا على الصحة النفسية بسبب قلة التفاعل الاجتماعي؟	القسم الثاني: المعرفة حول تأثير ألعاب الفيديو على العزلة الاجتماعية (6 أسئلة، بنعم / لا / لا أعلم)
هل لديك معرفة بأن ألعاب الفيديو لا يمكن أن تكون بديلًا كاملًا عن التفاعل الاجتماعي المباشر وجهًا لوجه؟	
هل لديك معرفة بأن الانغماس المفرط في الألعاب قد يؤدي إلى تدهور العلاقات الأسرية والاجتماعية؟	
هل لديك معرفة بأن من الآثار النفسية لقضاء وقت طويل في اللعب هو الشعور بالوحدة؟	
هل تعتقد أن عدم المشاركة في الأنشطة الاجتماعية بسبب ألعاب الفيديو قد يؤدي إلى العزلة؟	
هل لديك معرفة بأن ألعاب الفيديو قد تسبب العزلة نتيجة نقص التفاعل الاجتماعي؟	
هل لاحظت أن أصدقائك اللاعبين أصبحوا منعزلين أو يفضلون اللعب على المحادثات وجهًا لوجه؟	
إلى أي مدى توافق على العبارة: “يمكن أن تؤدي ألعاب الفيديو إلى العزلة الاجتماعية”؟	القسم الثالث : مستوى الاتفاق مع العبارات المتعلقة بالألعاب والعزلة الاجتماعية 10 أسئلة؛ مقياس ليكرت 5 نقاط: أوافق بشدة → أوافق → محايد → لا أوافق → لا أوافق بشدة)
إلى أي مدى توافق على العبارة: “ألعاب الفيديو طريقة لتجنب التفاعل الاجتماعي”؟	
إلى أي مدى توافق على العبارة: “من الجيد قضاء وقت طويل في لعب ألعاب الفيديو”؟	
إلى أي مدى توافق على العبارة: “هناك علاقة بين الوقت المخصص للعب الألعاب وسوء التفاعل الاجتماعي”؟	
إلى أي مدى توافق على العبارة: “الوقت المفرط في اللعب يقلل من فرص التفاعل المباشر مع الآخرين”؟	
إلى أي مدى توافق على العبارة: “اللعب المفرط يؤثر سلبًا على العلاقات الاجتماعية”؟	
إلى أي مدى توافق على العبارة: “يجب الحفاظ على توازن بين الحياة الاجتماعية ولعب ألعاب الفيديو”؟	
إلى أي مدى توافق على العبارة: “أخذ فترات استراحة طويلة من اللعب يحسن العلاقات الاجتماعية”؟	
إلى أي مدى توافق على العبارة: “العزلة الاجتماعية هي نتيجة طبيعية لإدمان ألعاب الفيديو”؟	
إلى أي مدى توافق على العبارة: “يمكن أن تسبب ألعاب الفيديو شعورًا بالعزلة حتى عند اللعب مع الآخرين عبر الإنترنت”؟	
هل تختار لعب ألعاب الفيديو بدلًا من قضاء الوقت مع الأصدقاء أو العائلة؟	القسم الرابع : السلوكيات المتعلقة بالألعاب والعزلة الاجتماعية (13 سؤالًا؛ مقياس ليكرت 4 نقاط: دائمًا → أحيانًا → نادرًا → أبدًا)
عندما تشعر بالوحدة، هل تميل إلى اللعب أكثر للتعامل مع هذا الشعور؟	
هل تعتقد أن التفاعل الاجتماعي داخل الألعاب يمكن أن يكون بديلًا للتفاعل الواقعي؟	
هل تقلق بشأن الوقت الذي تقضيه في اللعب وكيف يؤثر على علاقاتك الاجتماعية؟	
هل حاولت يومًا تقليل وقت اللعب لتحسين حياتك الاجتماعية؟	
إذا قمت بذلك، هل نجح هذا في تحسين علاقاتك الاجتماعية؟	
إلى أي مدى تميل لاختيار اللعب بدلًا من المحادثات وجهًا لوجه عندما يكون لديك وقت فراغ؟	
هل يؤثر الوقت الذي تقضيه يوميًا في اللعب على رغبتك في التفاعل مع الآخرين؟	
هل تجد صعوبة في المشاركة في الأنشطة الاجتماعية بالجامعة بسبب كثرة لعب الألعاب؟	
هل تميل إلى تجنب التجمعات الاجتماعية وجهًا لوجه لتبقى في المنزل وتلعب؟	
هل لاحظت انخفاضًا في دائرة معارفك الواقعية نتيجة لقضاء وقت أكثر في اللعب؟	
هل تعتقد أن ألعاب الفيديو هي وسيلتك الوحيدة للتواصل مع الآخرين؟	
هل فقدت بعض علاقاتك الاجتماعية بسبب انغماسك أو اهتمامك بالألعاب؟	

**Table 12:** Detailed coding and statistical procedures

Variable/scale	Original responses	Recoding/scoring	Purpose/notes		
Gaming time	≤4 hours/day, >4 hours/day	0 = ≤4 hrs, 1 = >4 hrs	Binary variable used in odds ratio analyses		
Preference for gaming over face-to-face interaction	Yes, no	1 = yes, 0 = no	Indicates social preference orientation		
Awareness of isolation	Yes, no	1 = yes, 0 = no	Measures awareness of potential isolation risk		
Belief in balance	Strongly agree, agree, neutral, disagree, strongly disagree	1 = agree or strongly agree, 0 = otherwise	Reflects belief in balanced lifestyle		
Isolation level score	Always, sometimes, rarely, never	Always = 3, sometimes = 2, rarely = 1, never = 0; total score range: 0–39	Composite variable; categorized as: 0–13 = low, 14–26 = moderate, 27–39 = high		
Attitudinal items (Section 4)	Strongly disagree → strongly agree (5-point scale)	1 = strongly disagree → 5 = strongly agree	Mean score interpretations: 1.00–1.80 = strong disagreement, 1.81–2.60 = disagreement, 2.61–3.40 = neutral, 3.41–4.20 = agreement, 4.21–5.00 = strong agreement		
Multiple-response questions	Multiple options per item	Frequency of each selected option converted to a percentage of total responses	Used to describe selection patterns among participants		
Software used	—	JMP Pro 18, Microsoft Excel 2021	Statistical analysis and data management		
Statistical tests	—	Chi-square test, Cramer’s V, odds ratios	Used to test associations and effect sizes between categorical variables		
